# Snare-assisted delivery and coaxial optimization of a self-expanding transcatheter aortic valve in the horizontal aorta

**DOI:** 10.1093/ehjcr/ytaf159

**Published:** 2025-04-05

**Authors:** Umihiko Kaneko, Daisuke Hachinohe, Ryo Horita, Ken Kobayashi

**Affiliations:** Department of Cardiovascular Medicine, Sapporo Cardiovascular Clinic, North 49, East 16, 8-1 Higashi Ward, Sapporo, Hokkaido 007-0849, Japan; Department of Cardiovascular Medicine, Sapporo Cardiovascular Clinic, North 49, East 16, 8-1 Higashi Ward, Sapporo, Hokkaido 007-0849, Japan; Department of Cardiovascular Medicine, Sapporo Cardiovascular Clinic, North 49, East 16, 8-1 Higashi Ward, Sapporo, Hokkaido 007-0849, Japan; Department of Cardiovascular Medicine, Sapporo Cardiovascular Clinic, North 49, East 16, 8-1 Higashi Ward, Sapporo, Hokkaido 007-0849, Japan

## Case description

**Figure 1:**
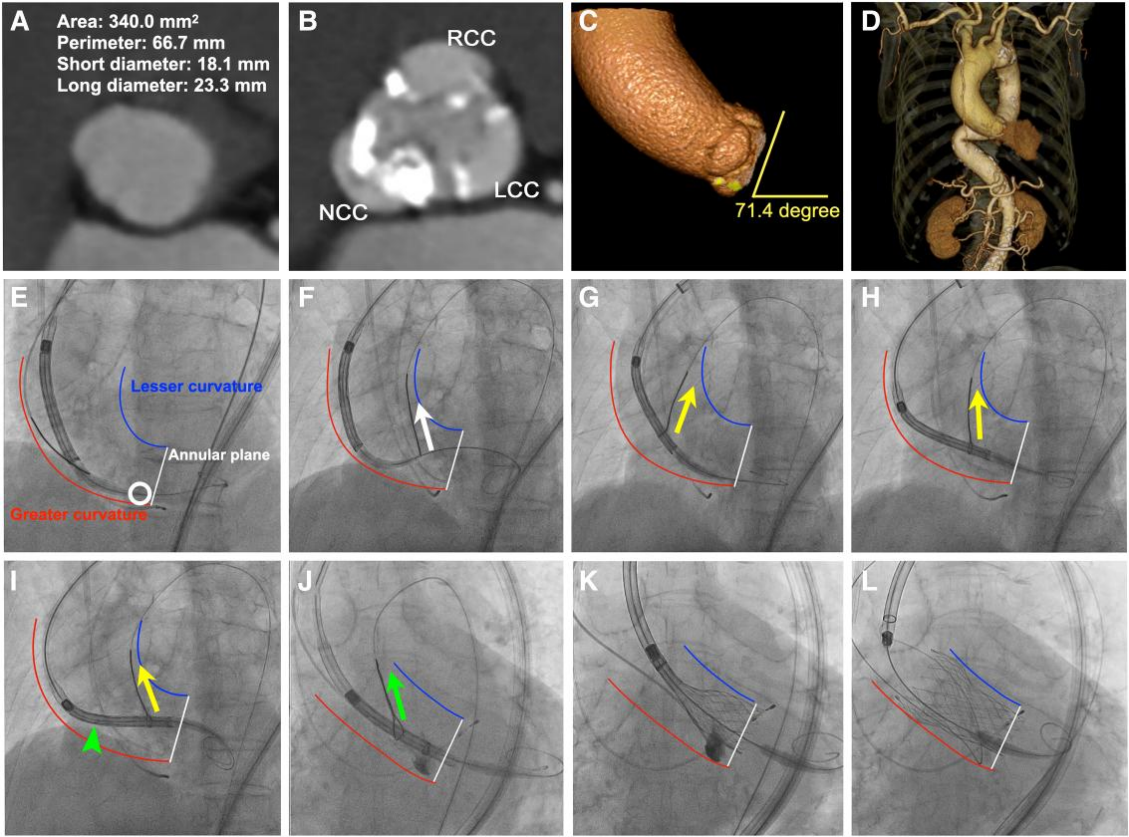
Snare-assisted delivery and coaxial optimization. (*A–D*) Pre-procedural computed tomography demonstrates severe calcification, particularly in the non-coronary cusp (NCC), along with an extremely horizontal ascending aorta (aortic root angle: 71.4°) and a tortuous descending aorta. (*E*) Bioprosthesis failed to cross the aortic valve because of extensive NCC calcification (white circle). (*F*) The nose cone was initially snared but failed to cross the aortic valve (white arrow). (*G–I*) Snare engagement at the lower third of the delivery capsule facilitated valve advancement (yellow arrows); however, non-coaxial alignment was observed (green arrowhead). (*J*, *K*) Snare engagement at the upper third of the capsule achieved proper coaxial alignment (green arrow). (*L*) Final result. White line: annular plane. Blue line: lesser curvature of the ascending aorta. Red line: greater curvature of the ascending aorta.

A woman in her 90s with symptomatic severe aortic stenosis (mean pressure gradient: 52 mmHg, aortic valve area by planimetry: 0.73 cm²) underwent transcatheter aortic valve replacement (TAVR) using a 26-mm self-expanding Evolut FX valve (Medtronic, Minneapolis, MN, USA). Computed tomography revealed severe calcification, particularly in the non-coronary cusp (NCC), an extremely horizontal aorta (aortic root angle: 71.4°), and a tortuous descending aorta (*Figure 1A–D*).

Following insertion of a 65-cm 18-F DrySeal sheath (W.L. Gore & Associates) into the ascending aorta over double Lunderquist guidewires (Cook Medical) and subsequent pre-dilation with a 20-mm balloon, the transcatheter heart valve (THV) was successfully delivered to the ascending aorta. However, initial attempts to cross the aortic valve with the THV were unsuccessful because of extensive NCC calcification (*Figure 1E* and Supplementary material online, *Video S1*). Moreover, efforts to grasp the nose cone with a 25-mm ONE Snare catheter (Merit Medical) from the contralateral femoral access failed to advance the THV (*Figure 1F* and Supplementary material online, *Video S2*). Subsequently, the snare was repositioned to the lower third of the delivery capsule, facilitating valve passage (*Figure 1G* and *H* and Supplementary material online, *Video S3*). Despite these manoeuvres, achieving coaxial alignment of the THV with the annulus remained impossible (*Figure 1I*); securing the upper third of the delivery capsule with the snare enabled successful coaxial deployment (*Figure 1J–L* and Supplementary material online, *Video S4*). Post-procedural echocardiography and aortography revealed a well-functioning valve with a mean gradient of 7 mmHg and mild paravalvular leak (see Supplementary material online, *Video S5*).

An extremely horizontal aorta is characterized by pronounced angulation of the ascending aorta relative to the annulus, often accompanied by significant tortuosity.^1^ These anatomical features can complicate THV delivery and hinder coaxial deployment. Placing a 65-cm large-calibre DrySeal sheath above the most tortuous segment of the aorta provides a simple and reliable approach to facilitate THV delivery.^2^ To our knowledge, this is the first report on the combined use of an ultra-long sheath and a snare technique specifically for cases with a highly horizontal and tortuous aorta.

While some reports suggest that snare techniques can facilitate THV delivery across the aortic valve, detailed technical guidance—such as the identification of optimal snaring locations and timing—remains limited.^3^ In this case, snaring the lower third of the delivery system—identified as the least coaxial site and obstructed by NCC calcification—enabled transaortic valve passage. However, the horizontal aortic anatomy severely hindered optimal coaxial alignment. This required repositioning the snare to the upper third of the delivery capsule—identified as the most off-axis location—to achieve successful coaxial deployment. Poorly coaxialized segments should be grasped with a snare to achieve coaxiality for both transaortic valve advancement and subsequent coaxial optimization.

## Supplementary Material

ytaf159_Supplementary_Data

## Data Availability

The data underlying this article are available in the article.

## Notes

**Consent:** The authors confirm that, in accordance with COPE guidelines, the patient has provided written consent for the submission and publication of this case report, including the accompanying text and images.

**Funding:** This study received no financial support.
